# The efficacy of computer reminders on external quality assessment for point-of-care testing in Danish general practice: rationale and methodology for two randomized trials

**DOI:** 10.1186/1748-5908-6-79

**Published:** 2011-07-23

**Authors:** Frans B Waldorff, Volkert Siersma, Ruth Ertmann, Marius Brostrøm Kousgaard, Anette Sonne Nielsen, Peter Felding, Niels Mosbæk, Else Hjortsø, Susanne Reventlow

**Affiliations:** 1 The Research Unit for General Practice and Section of General Practice, Department of Public Health, University of Copenhagen, Copenhagen, Denmark; 2 Section of General Practice and Research Unit for General Practice, Department of Public Health, University of Copenhagen, Copenhagen, Denmark; 3 Copenhagen General Practice Laboratory, Copenhagen, Denmark; 4 Capital Region, Hillerød, Denmark

## Abstract

**Background:**

Point-of-care testing (POCT) is increasingly being used in general practice to assist general practitioners (GPs) in their management of patients with diseases. However, low adherence to quality guidelines in terms of split test procedures has been observed among GPs in parts of the Capital Region in Denmark. Computer reminders embedded in GPs electronic medical records (ComRem) may facilitate improved quality control behaviour, but more research is needed to identify what types of reminders work and when. The overall aim of this study is to evaluate the efficacy of ComRem to improve GPs adherence to quality guidelines. This article describes the rationale and methods of the study that constitute this research project.

**Methods/design:**

The study is conducted as two randomised controlled trials (RCTs) among general practices in two districts of the Capital Region in Denmark. These districts contain a total of 739 GPs in 567 practices with a total of 1.1 million patients allocated to practice lists. In the first RCT (RCT A), ComRem is compared to postal reminder letters. In the second RCT (RCT B), ComRem is compared to usual activities (no reminders) with a crossover approach. In both of these studies, outcomes are measured by the number of split tests received by the laboratory.

**Conclusions:**

This study will contribute to knowledge on the efficacy of ComRem in primary care. Because the study does not explore GPs' perceptions and experiences with regard to ComRem, we will subsequently conduct a qualitative survey focusing on these aspects.

**Trial registrations:**

Study A: ClinicalTrials.gov identifier: NCT01152151

Study B: ClinicalTrials.gov identifier: NCT01152177

## Background

Point-of-care testing (POCT) is increasingly being used in general practice to assist general practitioners (GPs) in their daily work with patients. For adequate deployment of POCT, an external quality assessment (EQA) is recommended on a monthly basis [1]. In the Copenhagen area, EQA is enforced by a split test procedure as well as annual outreach consultant visits. In a split test, the actual POCT result is compared to a result from a blood sample from the same individual analyzed at the central laboratory. The quotient of these two results should ideally be 1.00, but a value inside the interval ranging from 0.85 to 1.15 is acceptable [1]. This quotient is returned to the practice for self-evaluation. However, the adherence to the monthly split test procedure has not been satisfactory among GPs in two districts of the Capital Region (Table 1). Therefore, the Copenhagen General Practitioners' Laboratory (hereafter simply referred to as 'the laboratory') planned to improve adherence.

**Table 1:** GP and practice characteristics and distribution of the Point of Care tests at baseline (January to April 2010) stratified on the two RCTs

		Study A	Study B
		**Computer reminder/postal reminder**	**Computer reminder/usual care**
		**(Practices = 213)**	**(Practices = 286)**
		**(GPs = 318)**	**(GPs = 341)**
		**Mean (SD) or n (%)**	**Mean (SD) or n (%)**

GP and practice characteristics			
Gender	Male	177 (56)	171 (50)
	Female	141 (44)	170 (50)
Mean age (SD)		53.1 (7.8)	56.3 (8.2)
Mean years as GP		11.3 (8.8)	14.8 (9.6)
Total number of patients on practice lists		507,568	533,864
Mean number of patients per GP (SD)		1,596 (419)	1,566 (398)
Practice organization	Single Handed	139 (65)	239 (84)
	Group	74 (45)	47 (16)
Point of Care Tests at baseline			
Mean INR tests		86 (58)	---------------------
Mean Hemoglobin tests		63 (97)	40 (55)
Mean Glucose tests		78 (92)	47 (52)
Primary outcome		INR*	HGB/GLU**
Average number of split tests		0.35 (0.55)	1.07 (1.36)
Secondary outcomes			
Practice with three or four split tests		0 (0)	0 (0)
Practices with at least one split test		67 (31)	146 (51)

*four recommended in baseline period

**four are recommended for each of the two tests in baseline period

Dissemination of guidelines alone rarely brings about improvements in clinical practice [2], and even an multifaceted implementation of guidelines may not change clinical practice [3,4]. Multiple strategies for implementing guidelines appear to be more effective than single ones [5,6]. However, when evaluated rigorously, these strategies often produce only minor benefits and rarely on patient outcomes [7,8]. In general, well-designed empirical research looking into various implementation strategies is still needed in this area [9]. In order to improve adherence, the laboratory wants to employ computer reminders embedded in the GPs' electronic medical records (ComRem) additionally to the quality control enforcement activities. At the same time, the laboratory wanted to conduct a rigorous evaluation of this innovative approach.

Within the last decade, several systematic reviews have evaluated computer reminders [10-13]. However, these reviews have pooled several different types of computer reminders, *e.g*., computer-generated paper reminders, and e-mail alerts along with reminders generated at point of care. According to a recent Cochrane review, computer reminders have been shown to produce a small improvement on target behaviour [13]. The review concludes that more research is needed to identify what types of reminders work and when.

The overall aim of the randomised controlled trials (RCTs) described in this paper is to evaluate the efficacy of ComRem on adherence to clinical quality guidelines regarding POCT. This aim is translated into the following research questions:

1. What is the efficacy of ComRem compared to computer-generated postal reminder letters on general practices' adherence to clinical quality guidelines regarding POCT (RCT A)?

2. What is the efficacy and legacy of ComRem compared to usual activities (*i.e*., no special reminders) on GP adherence to clinical quality guidelines regarding POCT (RCT B)?

The reason why the study is divided into two RCTs is the laboratory's priority to implement a quality improvement intervention for all practices conducting international normalized ratio (INR) analyses. Hence, these practices could not be allocated to usual practice alone.

## Methods

The study is conducted as two RCTs among the practices in two districts in the Capital Region in Denmark. These districts contain a total of 739 GPs in 567 practices.

### Usual EQA practice

The guidelines recommend a split test procedure each month for each POCT instrument. The standard implementation of EQA consists of an annual facilitator visit in each practice. Also, information about the split tests was posted in three laboratory newsletters. Furthermore, an instruction on how the practices should execute split tests was posted as a link on the laboratory's web site.

### Computer reminders embedded in the GPs' electronic medical records (ComRem)

In the Danish health care sector, a common standard (MedCom) for electronic communication has been defined and used for a decade. MedCom is a co-operative venture between authorities, organizations, and private firms linked to the Danish healthcare sector, and uses a Danish adapted format of Electronic Data Interchange For Administration, Commerce and Transport (UN/EDIFACT) originally developed by the United Nations. MedCom offers secure electronic communication between hospitals, pharmacies, laboratories, and primary care, *e.g*., GPs and municipalities. Each practice has been allocated to a unique location number according to the MedCom standard. All electronic communication is delivered into a specific practice inbox and each communication has to be approved by the GP.

### Participants

Included in the study are all general practices that had performed at least four relevant POCT--INR, hemoglobin, or glucose--in the baseline study period (1 January to 31 April 2010) and had access to POCT within their own practice. These practices were identified in the GP database of the Capital area and in the laboratory database.

### Data collection

Data on performed split test procedures is retrieved from the laboratory database. These data do not contain any patient-related data because all split tests are conducted by an artificial identification code. Process indicators (sent reminder letters) are also obtained from the laboratory. The Capital Region databases provide information on the participating practices and corresponding GPs.

### Randomization

Within each study, practices are randomized into two similarly sized groups by means of computer generated random numbers using SAS version 9.2. To ensure that practice types are distributed equally over the intervention and control arms, randomization within each RCT is done separately for group practices and solo practices respectively. This randomization was conducted by the data manager of the Research Unit of General Practice without knowledge of the individual practice identification.

### Data from the capital region and Copenhagen general practice laboratory

The study period comprises a four-month baseline period before the start of the intervention and up to three four-month follow-up periods after the start of the intervention. During the study periods, the number of specific POCT tests done in each practice is monitored using the relevant bills sent to the Capital Region by the GPs. Concurrent, the occurrence of split tests on the specific POCT analyses and--for practices participating in an intervention arm--the number of ComRem or postal reminders sent in each of the follow-up periods are monitored in the laboratory database. Combined, this monitoring gives series of up to four measures (defined by several outcomes, see below) of adherence to the split test procedures--for the baseline period and for each of the follow-up periods--for each practice. Background information on practices and GP was retrieved by the Capital Region at the start of the study period. This information includes gender, age, year of graduation from university, working address, type of practice, and list size.

### Outcomes

Outcomes are measures of split test procedure adherence that are calculated from the monitor data.

### Primary outcome

Total number of split test procedures for the corresponding POCT analysis performed by the practice in a four-month period. In a given month, a split test procedure should only be performed if the practice conducted the POCT analysis. The maximum number of possible split test procedures for a single analysis in a four-month period is four.

### Secondary outcomes

1. Whether the practice has a high quality of tests defined as 75% of the performed split test procedures within the reference interval (according to the laboratory quality guidelines).

2. Whether split test procedures are performed at all by the practice.

### Statistics

Cross-sectional differences in the outcomes between allocation groups in the baseline period and each of the follow-up periods are tested by means of t-tests (primary outcome) and chi-square tests (secondary outcomes). In order to investigate the development of split test procedure adherence over the up to four periods relative to the (changing) intervention, Poisson (primary outcome) and logistic (secondary outcomes) regression is used with GEE methods to account for the repeated measurements. To identify predictors for adhering to split test procedure guidelines, adjusted effects for the GP and practice characteristics are estimated in multivariate Poisson (primary outcome) and logistic (secondary outcomes) regression analysis on the outcomes at selected periods. Beyond the direct comparison of the randomization groups in the first follow-up period, comparisons in further follow-up periods are done to investigate various forms of potential legacy effect, *i.e*., the effect of an intervention in periods where the intervention is discontinued. All statistical analyses are performed using SAS, version 9.2 (SAS Institute Inc, Cary, NC).

### RCT A: Comparing computer reminders with postal reminders

Included in RCT A are the practices conducting more than four POCT INR during the four-month baseline period (213 practices). Practices are randomly allocated to computer-generated postal reminder letters or to ComRem; both in addition to usual laboratory quality guideline activities. The comparison of outcomes after a first follow-up period: the intervention period estimates the relative efficacy of ComRem over postal reminder letters; a comparison after a second follow-up period in which both interventions are discontinued estimates the relative legacy effect of the interventions.

## Intervention

### Postal reminder letters

**Figure 1:**
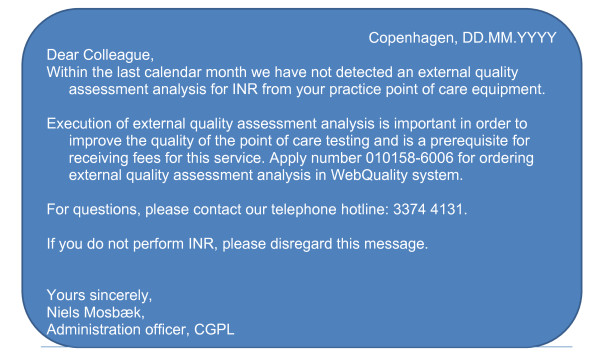
**Contents of reminder (RCT A)**.

In this group, computer-generated postal reminder letters are sent to practices not adhering to the guideline recommendations of split testing within the previous calendar month. Thus, practices may receive up to four postal reminder letters during each four-month follow-up round. The contents of the reminder are presented in Figure 1.

### ComRem

In this group, ComRem are sent to practices not adhering to the guideline recommendations of split testing within the previous calendar month. Thus, practices may receive up to four ComRem. These reminders have exactly the same content as the postal reminders.

### Outcomes

The outcomes specified previously address the conduct of POCT for INR. The study period comprises three four-month periods: The baseline, the intervention, and follow up period.

### Power calculation

We used an estimate of a mean number of 1.5 INR split tests based on laboratory data from 2007 in order to ascertain the power of the study. Given a standard deviation of 1, a power of 90%, and an effect of 0.5, we estimated that 172 practices are to be included in this study.

### RCT B: Comparing computer reminders with usual activities and the potential legacy of electronic reminders

Included in the study are the 286 general practices conducting at least four POCT (either hemoglobin or glucose) during the four-month baseline period and that are not already included in RCT A. Practices are allocated to ComRem together with usual laboratory EQA practice or usual laboratory EQA practice only. The comparison of outcomes after a first follow-up period, the intervention period, estimates the relative efficacy of ComRem versus usual care only; a comparison after a second follow-up period in which the interventions are switched between groups estimates the relative efficacy of a (short-term) legacy effect of ComRem over the direct effect of ComRem; a comparison after a third follow-up period in which both interventions are discontinued estimates the relative efficacy of a long-term legacy effect of ComRem over the short-term legacy effect of ComRem.

## Intervention

### ComRem

Computer reminder letters are sent to practices not adhering to the guideline recommendations of split testing for either hemoglobin or glucose within the previous calendar month. Thus, all practices may receive up to four electronic reminder letters in each period. The content of the reminder is identical to the one in RCT A, except that this reminder addresses hemoglobin/glucose and not INR.

### Outcome

The outcomes specified previously address the conduct of POCT for hemoglobin and glucose. The study period comprises four four-month periods: The baseline, the intervention, cross-over, and follow up period.

### Power calculation

We used an estimate of a mean number of one hemoglobin and/or glucose split test based on laboratory data from 2007 in order to ascertain the power of the study. Given a standard deviation of 1.25, a power of 90% and an effect of 0.5, we estimated that 266 practices are to be included in this study.

### Ethics

This study uses blood samples that are not retraceable to specific patients in order to conduct the split tests. Patient identities are kept hidden through an artificial identification code used by all practices. The project has been evaluated by the Scientific Ethical Committee for Copenhagen and Frederiksberg Municipalities (j. nr. H-1-2010_FSP/10) and the Danish Data Protection Agency (j. nr. 2010-41-4680). The Danish College of General Practitioners Study Committee evaluated the project (MPU 12-2010). The two RCT are registered in ClinicalTrials.gov Identifier (NCT01152151, NCT01152177).

## Results

**Figure 2:**
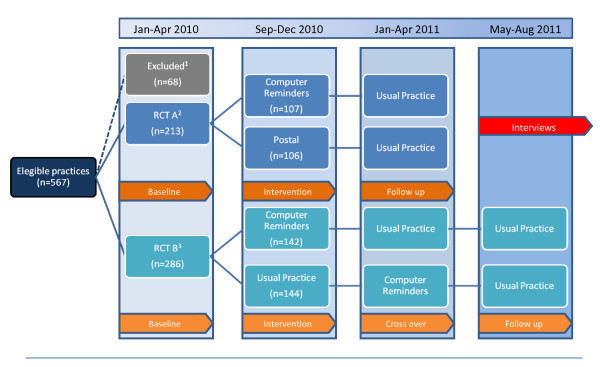
**Trial flow for the project**.

A total of 567 practices with a total of 739 GPs were eligible in the study area with a total population of 1.1 million. In total, 213 practices were included in RCT A and 286 were included in RCT B (Figure 2). The characteristics of GPs and practices are presented in Table 1. Split tests were in general infrequently used by the practices (Table 1).

## Discussion

This paper describes the rationale and design of two RCTs testing the efficacy of ComRem on GPs' adherence to quality guidelines on POCT. Thus, our study is in accordance with a recent Cochrane Review that calls for more research into ComRem in order to identify the features associated with improvements in provider behavior [13].

This is the first study in general practice to address computer reminders. All practices in the two districts are eligible, and only practices with few POCT or with no POCT equipment are excluded. Thus, the study population represents behavior among those who routinely use POCT in their clinical decisions. The two RCTs studies are designed to comply with the recommendations of the CONSORT statement [14]. The data manager conducted the randomization without knowing the practice code. The participating practices are unaware of their randomization group allocation up to the point when the first reminder (electronic or postal) does or does not come in. Because the study is incorporated in the activities of the laboratory, many practices may not realize that they are participating in the RCTs. Thus, the results from this study represent the present daily standard. Blinded assessment of study outcomes is not relevant, because outcome data is retrieved from the laboratory's electronic data system that documents delivery of the split tests as well as routine data from the Capital Region [13]. Because not much is known on the efficacy of ComRem on POCT quality, it was difficult to do a power analysis in the design phase of the studies. Hence, we based the power analysis on a laboratory report from 2007 and experiences with postal reminders [15].

In the two RCTs we evaluate different approaches. In RCT A we compare computer reminders with postal reminders, and in RCT B we compare computer reminders with usual activities. The two RCTs are conducted simultaneously, without any redundancy between practices. The Capital Region and the laboratory had beforehand agreed to focus on INR. Due to the seriousness of producing a wrong INR analysis result, it was demanded that both arms should have an intervention involving prompting, because any prompting most likely would increase the adherence rate. Hence, no comparison with usual laboratory EQA practice only was done in RCT A. RCT B offers the possibility to survey a legacy effect, which is how long one should expect an effect of computer reminders to last. This aspect has not previously been studied. In addition to the main aims, we will have the possibility to measure cost effectiveness as well as indirectly derived efficacy on other POCT analyses *e.g*., the effect on c-reactive protein split tests.

The EQA procedures for POCT here described reflect the standard in Denmark. Other countries' EQA procedures for POCT may not use prompting, and our study's results do not generalize to them. However, POCT may be viewed as a case of more general prompting, and our study's results may be generalized to other systems where prompting is used.

In order to advance our interpretation of the results and our understanding of how the electronic reminders relate to beliefs, technologies, and routines in general practice, a qualitative study using semi-structured interviews [16] will be conducted after the two RCTs are completed. Linking qualitative methods to RCT studies is increasingly recommended in the field of health services research [17] where qualitative methods have been applied before, during or after randomized controlled trials [18]. The key questions to be explored in the planned qualitative study are: How is quality control concerning POCT organized in the clinics and how do the GPs perceive the request for quality assurance for POCT? How do GPs regard the use of ComRem to promote adherence to quality assurance guidelines? What were the considerations and responses in the clinics (*e.g*., changes in procedures, roles, and responsibilities) following the reception of a ComRem? And how can variations in responses to ComRem be explained? Respondents for the interviews will be strategically selected [19] based on the results concerning the primary outcomes of the intervention (change versus no change in the number of split tests performed after ComRem).

On the basis of the quantitative and qualitative analysis, it will be considered if and how adjustments can be made to the content and employment of ComRem in order to support the ambitions of EQA in balance with other legitimate concerns of general practice.

In summary, research into computer reminders has been called for by a recent Cochrane review, and the present study focuses on primary care. This study will generate new knowledge on the efficacy and limitations of computer reminders in primary care.

## Competing interests

This survey was initiated by FBW, who is a GP in the study area. PF and NM are working in the Laboratory providing data and EH is a director in the Capital Region responsible for the reimbursement.

## Authors' contributions

FBW initiated the survey, designed and managed the efficacy trials, contributed to the qualitative survey and wrote the first manuscript draft. VS designed the efficacy trials and will do the statistical analysis. NM and PF designed the efficacy trials, provided data and manage the reminder procedures. EH designed the efficacy trials and provide data for the study. RE, MBK, ASN and SR participated in the design of the efficacy trials and will design and analyze the data in the qualitative survey. All authors have read and approved the final manuscript.

## End Notes

^1^Practices not conducting neither four INR tests nor four Hemoglobin/Glucose test in jan-april 2010 or did not have POCT; ^2^Practices conducting four or more INR test in jan-april 2010; ^3^Not conducting four INR test, but more than four Hemoglobin/Glucose test in jan-april 2010.
